# Metabolic profiling identifies potential biomarkers associated with progression from gestational diabetes mellitus to prediabetes postpartum

**DOI:** 10.7555/JBR.38.20240267

**Published:** 2024-11-08

**Authors:** Lenan Liu, Qian Yang, Panyuan Shen, Junsong Wang, Qi Zheng, Guoying Zhang, Bai Jin

**Affiliations:** 1 School of Public Health, Nanjing Medical University, Nanjing, Jiangsu 211166, China; 2 Department of Obstetrics, the First Affiliated Hospital of Nanjing Medical University, Nanjing, Jiangsu 210029, China; 3 Center of Molecular Metabolism, Nanjing University of Science and Technology, Nanjing, Jiangsu 210094, China

**Keywords:** metabolomics, gestational diabetes mellitus, follow-up, prediabetes, UHPLC-Q-TOF-MS/MS, impaired glucose tolerance

## Abstract

The current study aims to identify potential metabolic biomarkers that predict the progression to prediabetes in women with a history of gestational diabetes mellitus (GDM). We constructed a prediabetes group (*n* = 42) and a control group (*n* = 40) based on a 2-h 75 g oral glucose tolerance test for women with a history of GDM from six weeks to six months postpartum, and collected their clinical data and biochemical test results. We performed the plasma metabolomics analysis of the subjects at the fasting and 2-h post-load time points using ultra-high performance liquid chromatography-quadrupole time-of-flight mass spectrometry (UHPLC-Q-TOF-MS/MS). We found that the prediabetes group was older and had higher 2-h post-load glucose levels during pregnancy than the control group. The metabolomic analysis identified 164 differential metabolites between the groups. Compared with the control group, 15 metabolites in the prediabetes group exhibited consistent change trends at both time points, including three increased and 12 decreased metabolites. By building a prediction model of the progression from GDM to prediabetes, we found that a combination of three clinical markers yielded an area under the curve (AUC) of 0.71 (95% confidence interval [CI], 0.60–0.82). We also assessed the discriminative power of the panel of 15 metabolites for distinguishing between postpartum prediabetes and normal glucose tolerance of the subjects at the fasting (AUC, 0.98; 95% CI, 0.94–1.00) and 2-h post-load (AUC, 0.99; 95% CI, 0.97–1.00) time points. The metabolic pathway analysis indicated that energy metabolism and branched-chain amino acids played a role in prediabetes development in women with a history of GDM during the early postpartum period. In conclusion, this study identified potential metabolic biomarkers and pathways associated with the progression from GDM to prediabetes in the early postpartum period. A panel of 15 metabolites showed promising discriminative power for distinguishing between postpartum prediabetes and normal glucose tolerance. These findings provide insights into the underlying pathophysiology of this transition and suggest the feasibility of developing a metabolic profiling test for the early identification of women at high risk of prediabetes following GDM.

## Introduction

Gestational diabetes mellitus (GDM) complicates approximately 14% of pregnancies globally, with a prevalence of about 21% in Asia^[1]^. Women with a history of GDM face a seven to 10-fold increased risk of developing type 2 diabetes mellitus (T2D) and a two to three-fold higher risk of cardiovascular disease, compared with those without GDM^[2–4]^. Current guidelines (American Diabetes Association, 2024) recommend that all women with GDM undergo an oral glucose tolerance test (OGTT) at four to 12 weeks postpartum^[5]^. Intensive lifestyle modifications and metformin treatment have demonstrated a 35%–40% risk reduction in diabetes progression risk post-GDM compared with placebo^[6]^. Thus, identifying prediabetes early in these women is crucial for enabling interventions to delay or prevent the progression to diabetes.

However, global postpartum OGTT compliance averages only 33%, ranging between 9% and 71%^[7]^. Diabetes was reported in 2.8%–58% of the women based on the length of follow-up, while the prevalence of prediabetes, which is an intermediate stage between normal glucose regulation and diabetes, was 3.9%–50.9% in the early postpartum period^[8]^. OGTT has an accuracy rate of only 60%–70% to predict future T2D^[9]^. Factors such as the inconvenience and inaccuracy of the OGTT further contribute to the low follow-up rates. Thus, there is a pressing need to develop more convenient and precise reclassification methods for postpartum assessment because of the currently limited options.

Metabolomics offers a valuable approach to identifying metabolic changes associated with GDM and T2D. Several studies have suggested that circulating metabolites in the early postpartum period may predict T2D in women with a history of GDM^[9–11]^. However, most of these studies have focused on fasting plasma or serum and have rarely addressed prediabetes, which is more common than T2D among women with a previous history of GDM in the early postpartum period.

Prediabetes is a heterogeneous glucose metabolism disorder characterized by impaired fasting glucose (IFG) and impaired glucose tolerance (IGT), assessed by using a 2-h post-load glucose test. It is important to note that plasma/serum circulating metabolites are significantly influenced by diet and glucose load. Thus, we measured numerous metabolites in stored frozen plasma samples collected between six weeks and six months postpartum from women with a history of GDM, using the 2-h 75 g OGTT, at both fasting and 2-h post-load time points.

Our primary aim was to gain insights into the underlying pathophysiology of the transition from GDM to prediabetes in the early postpartum period through a metabolic approach under fasting and glucose load conditions, respectively. A secondary goal was to identify metabolic biomarkers of prediabetes for building a more convenient and accurate screening method in the future, to further develop strategies for cost-effective post-GDM monitoring.

## Materials and methods

### Study design

We conducted a cross-sectional study of women with a history of GDM based on the 2-h 75 g OGTT during pregnancy. Women who delivered a singleton, live-born infant at the First Affiliated Hospital of Nanjing Medical University between April 2021 and May 2022 were recruited. Women with multiple gestations, pre-gestational diabetes, severe health conditions, or medication use affecting glucose tolerance were excluded from the study. Eligible women with a history of GDM were enrolled in the study and underwent the 2-h 75 g OGTT between six weeks and six months postpartum. As a result, 40 women had normal postpartum glucose tolerance (the control group), and 42 women were diagnosed with postpartum prediabetes (the prediabetes group), including seven with IFG, 31 with IGT, and four with both IFG and IGT. Diagnostic criteria for GDM and prediabetes were based on the criteria of the International Association of Diabetes and Pregnancy Study Groups (IADPS) and the American Diabetes Association (ADA), respectively^[12]^. Given the poor concordance between the OGTT and glycosylated hemoglobin for diagnosing diabetes and prediabetes following GDM^[13–14]^, we only used the OGTT results as the diagnostic criteria for prediabetes in the current study.

Clinical data were collected from both patient visits and electronic medical records, including age, height, family history of diabetes, antepartum and postpartum laboratory results, pre-pregnancy weight, pre-delivery weight, postpartum weight, blood pressure, dates of diabetes diagnosis, and other clinical outcomes.

The area under the curve (AUC) of the OGTT time-blood glucose was calculated using the trapezoidal rule. Postpartum weight retention (PWR) was defined as the difference between postpartum weight and pre-pregnancy weight, while postpartum weight loss (PWL) was defined as the difference between postpartum weight and pre-delivery weight.

The current study protocol was approved by the Research Ethics Committee of the First Affiliated Hospital of Nanjing Medical University (Approval No. 2023-SR-579). Before enrollment, we obtained written informed consent from each subject.

### Plasma sample collection and preparation

Blood samples were collected from each participant at both fasting and 2-h post-load time points during the 75 g OGTT. The samples were centrifuged (1000 *g*, 10 min, 4 ℃) and then stored at −80 ℃ for subsequent metabolomics analysis.

### Metabolomics methods

#### Sample preparation for ultra-high performance liquid chromatography-quadrupole time-of-flight mass spectrometry (UHPLC-Q-TOF-MS/MS)

The pooled plasma, mixed with an equal aliquot of plasma sample from the control and prediabetes groups, was used as a quality control sample. Both plasma and quality control samples were mixed with four times the volume of pre-cooled methanol, vortexed for 30 s, frozen at −20 ℃ for 1 h, and centrifuged at 16000 *g* at 4 ℃ for 15 min. The supernatant was lyophilized to dryness, and then dissolved in an acetonitrile-water solution (1∶1, v/v), vortexed, and centrifuged again. Subsequently, 80 μL of the supernatant was transferred for UHPLC-Q-TOF-MS/MS analysis (AB Sciex, Framingham, MA, USA). The quality control samples were included in every 10 samples to guarantee instrument stability. The solvent in which the samples were dissolved was used as a blank to subtract the solvent peaks and eliminate systemic impurity signals. The starting and ending blank samples of each batch were examined to find any carryover or contamination.

#### UHPLC-Q-TOF-MS/MS analysis and data pre-processing

Chromatographic analysis was performed on a SCIEX ExionLC UHPLC system (AB Sciex) using an Atlantis Premier BEH C18 AX column (2.1 mm × 100 mm, 1.7 μm) (Waters, Milford, MA, USA) with a guard column (VanGuard FIT, Waters) for chromatographic separation. The column temperature was 40 ℃, and the injection volume was 5 μL. Gradient elution was carried out at a flow rate of 0.4 mL/min with 0.1% formic acid (Merck, Germany) in water (solvent A) and acetonitrile (Merck) (solvent B): 0–1 min, 1% B; 1–10 min, 1%–99% B; 10–13 min, 99% B; 13–14 min, 99%–1% B; and 14–17 min, 1% B.

Data acquisition in both positive and negative ion modes was performed using a TripleTOF 5600+ system (AB Sciex) equipped with a DuoSpray Source (electrospray ionization [ESI]; AB Sciex). The ESI conditions were set as follows: ion source gas 1 at 55 psi, ion source gas 2 at 55 psi, curtain gas at 35 psi, and spray voltage at 5.5 kV (+)/−4.5 kV (−). The time-of-flight mass spectrometry information-dependent acquisition mass spectrometry (TOF MS-IDA-MS/MS) acquisition included a TOF MS scan and a product ion scan based on information-dependent acquisition (IDA). The TOF MS scan range was 50–1000 m/z, and the TOF IDA-MS/MS (product ion) scan range was 40–1000 m/z. IDA mode settings were: declustering potential of 80 V (+)/−80 V (−), collision energy of 35 (± 15) V (+)/−35 (± 15) V (−). Dynamic background subtraction was applied to match the IDA tests for UHPLC-Q-TOF-MS/MS. A calibration delivery system was used for automatic calibration of TOF MS and TOF MS/MS every five samples.

Data acquisition was performed using Analyst TF 1.8.1 (AB Sciex), and then the raw data (.wiff files) were converted into the. mzML format using ProteoWizard software (Vanderbilt University, Nashville, TN, USA). The XCMS software (Scripps Research Institute, La Jolla, CA, USA) was used for peak alignment, retention time correction, and peak area extraction.

### Statistical analysis

The significance statistics were performed with SPSS Statistics version 26 (IBM, Armonk, USA), including the Chi-square test for categorical variables (*n* and %), Student's *t*-test for normally distributed continuous variables (mean and standard deviation), and the Mann-Whitney test for non-normally distributed variables (median and interquartile ranges).

The univariable and multidimensional statistical analyses were conducted using the R version 4.4.1 (R Core Team; https://cran.r-project.org) to detect significantly differential metabolites between the control and prediabetes groups at the fasting and 2-h post-load time points. The unsupervised principal component analysis (PCA) and supervised partial least squares discriminant analysis (PLS-DA) were used to visualize group patterns and calculate variable importance in projection (VIP) values for the metabolites. Metabolic pathway analysis and metabolite set enrichment analysis were carried out on the MetaboAnalyst 6.0 platform (https://www.metaboanalyst.ca/MetaboAnalyst/home.xhtml; accessed on July 18, 2024). A network diagram illustrating the interactions between metabolites and genes was generated using Cytoscape software (version 3.10.2; https://cytoscape.org). We performed the plotting of the receiver operating characteristic (ROC) curves and compared the AUCs using the SigmaPlot 14.0 (Systat Software, San Jose, CA, USA). Differences with a *P* < 0.05 were considered statistically significant.

## Results

### Demographic and clinical characteristics

The demographic characteristics of the subjects in both groups are summarized in ***Table 1***. The subjects were older in the prediabetes group than those in the control group (*P* = 0.010). There were no significant differences in terms of parity, family history of diabetes, feeding patterns, postpartum weight, PWL, PWR, postpartum blood pressure, postpartum waist circumference, and postpartum hip circumference between the control and prediabetes groups.

**Table 1:** Postpartum demographic characteristics of the subjects

Characteristics	Control (*n*=40)	Prediabetes (*n*=42)	*P*-value
Age [years, median (Q1, Q3)]	31 (29–33)	33 (30–35)	0.010^*^
Multipara [*n* (%)]	8 (20.0)	16 (38.1)	0.072
Family history of diabetes [*n* (%)]	17 (42.5)	17 (40.5)	0.852
Exclusive breastfeeding [*n* (%)]	20 (50)	16 (38.1)	0.278
Weight (kg, mean±SD)	60.4±9.0	61.4±7.2	0.594
PWL (kg, mean ± SD)	8.3±3.0	8.6±2.8	0.572
PWR (kg, mean±SD)	3.5±5.0	3.4±3.8	0.984
BMI (kg/m^2^, mean ± SD)	23.0±3.1	23.4±2.5	0.612
SBP (mmHg, mean±SD)	112±10	112±12	0.959
DBP (mmHg, mean ± SD)	70±7	71±9	0.546
Waist circumference (cm, mean±SD)	82.1±8.4	82.9±9.2	0.698
Hip circumference (cm, mean ± SD)	94.7±5.9	96.0±6.1	0.308
Waist-hip ratio (mean±SD)	0.87±0.05	0.86±0.06	0.718
Data are expressed as means ± standard deviation (SD), medians (interquartile ranges), or numbers (percentages). Differences between the two groups were tested by the Chi-square test for categorical variables, Student's *t*-test for normally distributed continuous variables, and the Mann-Whitney test for non-normally distributed variables. ^*^P < 0.05. Abbreviations: PWL, postpartum weight loss; PWR, postpartum weight retention; BMI, body mass index; SBP, systolic blood pressure; DBP, diastolic blood pressure.			

**Figure 1:**
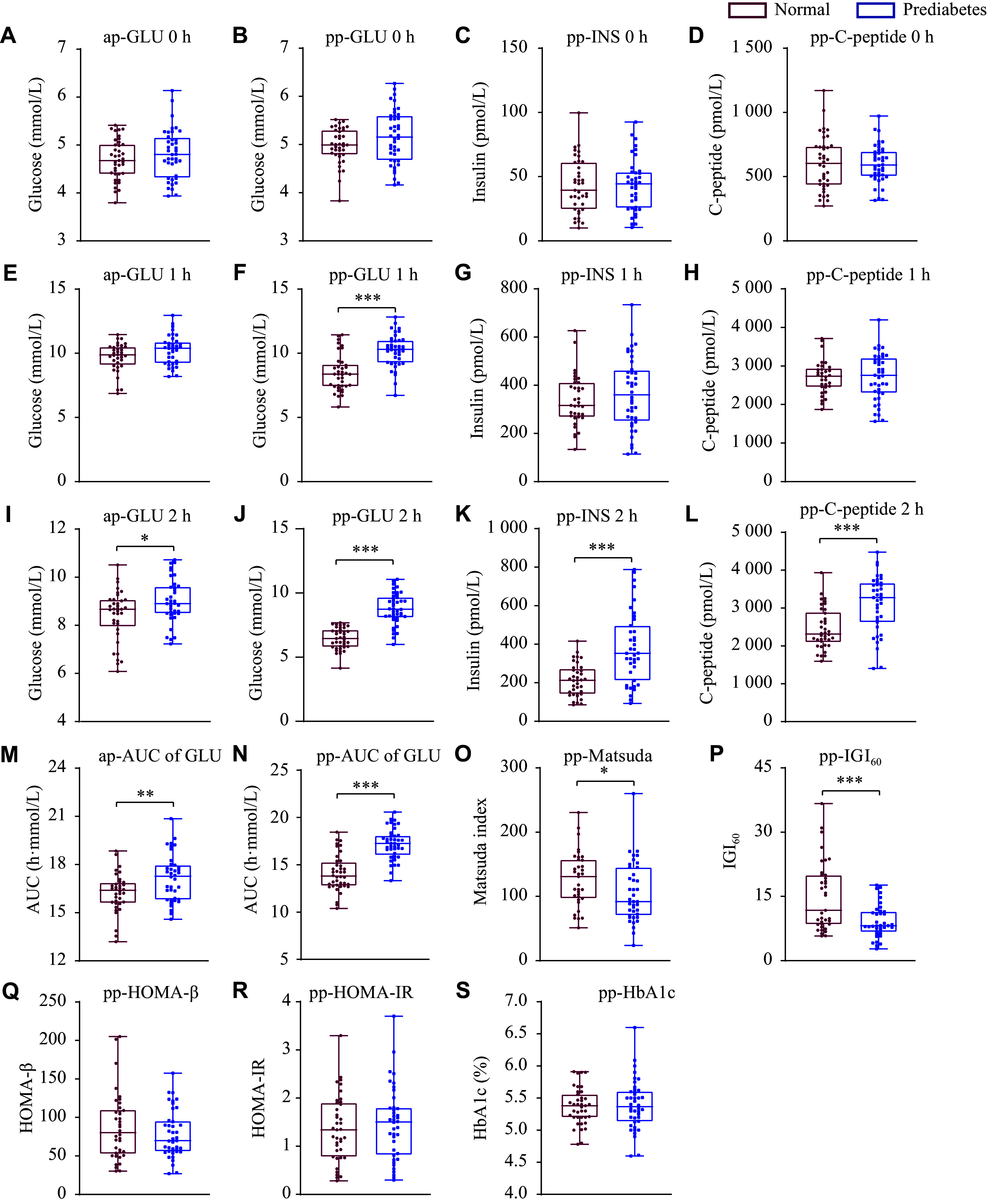
Box plots comparing the oral glucose tolerance test (OGTT) results and clinical characteristics during pregnancy and postpartum between the control (*n* = 40) and prediabetes (*n* = 42) groups. The top and bottom of the box plot represent the 25th and 75th percentiles, and the line across the box shows the median. The top and bottom bars show the maximum and minimum values. A: Glucose levels during the pregnancy OGTT at the fasting time point (0 h). B–D: Levels of glucose (B), insulin (C), and C-peptide (D) during the postpartum OGTT at the fasting time point (0 h). E: Glucose levels during the pregnancy OGTT at the 1-h post-load time point. F–H: Levels of glucose (F), insulin (G), and C-peptide (H) during the postpartum OGTT at the 1-h post-load time point. I: Glucose levels during the pregnancy OGTT at the 2-h post-load time point. J–L: Levels of glucose (J), insulin (K), and C-peptide (L) during the postpartum OGTT at the 2-h post-load time point. M and N: The AUCs of the time-blood glucose during the pregnancy OGTT (M) and postpartum OGTT (N). O–S: Postpartum levels of Matsuda index (O), IGI_60_ (P), HOMA-β (Q), HOMA-IR (R), and HbA1c (S). Data are presented as median and interquartile range. *P*-values were determined by the Mann-Whitney test. ^*^P < 0.05, ^**^P < 0.01, and ^***^P < 0.001. Abbreviations: ap, antepartum; pp, postpartum; GLU, glucose; INS, insulin; IGI_60_, insulinogenic index at 60 min; AUC, area under the curve; HOMA-β, homeostatic model assessment for beta cell; HOMA-IR, homeostatic model assessment for insulin resistance.

As shown in ***Fig. 1***, at the fasting time point, there were no significant differences in blood glucose levels during pregnancy and postpartum, as well as postpartum insulin and C-peptide levels, between the control and prediabetes groups (***Fig. 1A***–***1D***). At one hour after oral glucose administration, only the levels of blood glucose postpartum were significantly increased in the prediabetes group, compared with the control group, and there was no difference in postpartum insulin, C-peptide, and blood glucose levels during pregnancy (***Fig. 1E***–***1H***). However, two hours after oral glucose administration, the levels of blood glucose, insulin, and C-peptide during both pregnancy and postpartum were significantly elevated in the prediabetes group, compared with the control group (***Fig. 1I***–***1L***).

The AUCs of the OGTT time-blood glucose curve were significantly greater in the prediabetes group than those in the control group, during both pregnancy and postpartum periods (***Fig. 1M*** and ***1N***).

Compared with those in the control group, the subjects in the prediabetes group showed both a lower Matsuda index (*P* = 0.025) and a lower insulinogenic index at 60 min (*P* = 0.012; ***Fig. 1O*** and ***1P***). There were no significant differences in the homeostatic model assessment for beta-cell function (***Fig. 1Q***), the homeostatic model assessment for insulin resistance (***Fig. 1R***), and glycosylated hemoglobin levels (***Fig. 1S***) between the two groups.

### Metabolome spectrum for UHPLC-Q-TOF-MS/MS

**Figure 2:**
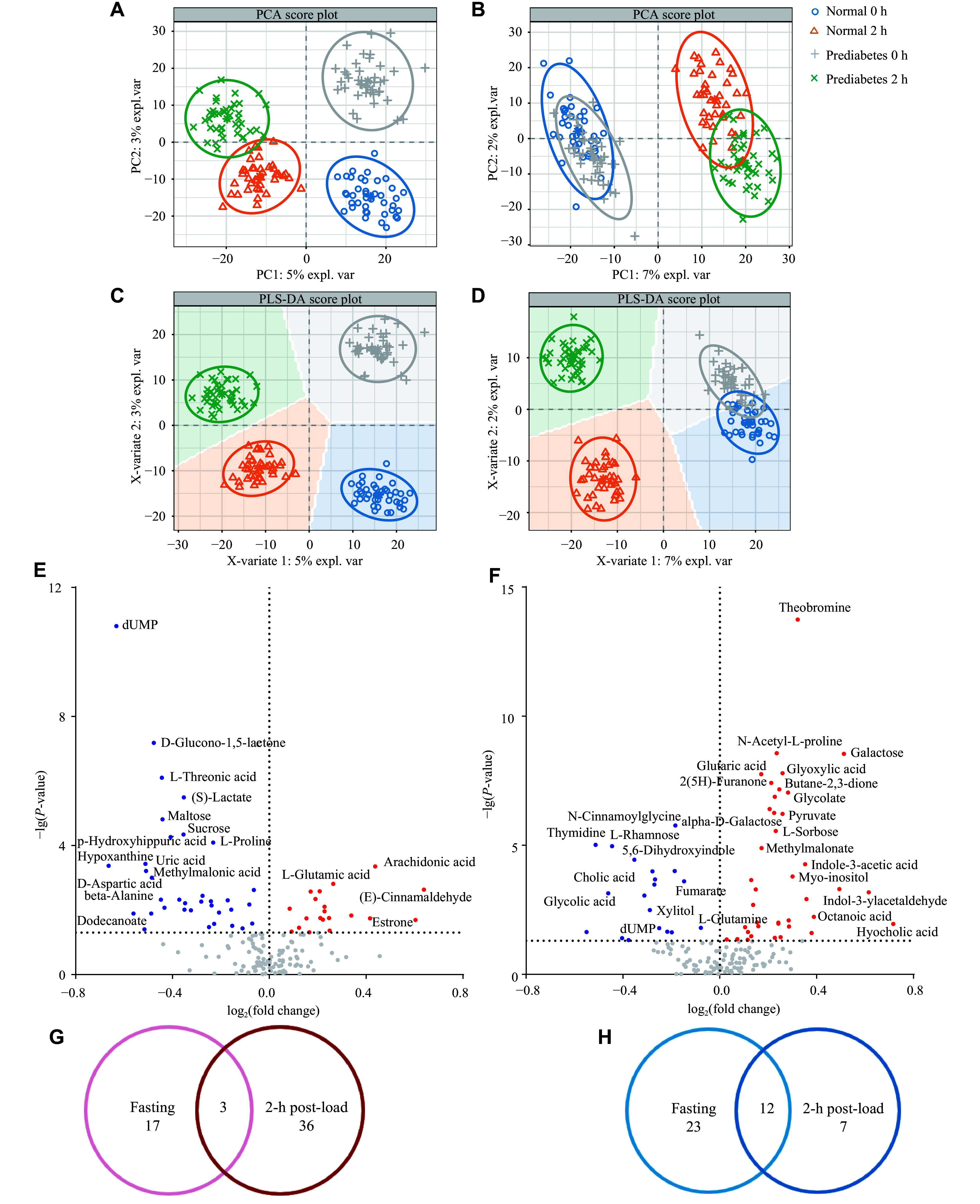
The PCA and PLS-DA score plots of each group and metabolites with differences between the control (*n* = 40) and prediabetes (*n* = 42) groups at fasting and 2-h post-load time points. A–D: The PCA and PLS-DA score plot for negative (A and C) and positive (B and D) ion modes, respectively. Each point represents a sample. Different groups are shown in different colors, with circles representing the 95% confidence intervals. E–F: Volcano plots of metabolites between groups at the fasting (E) and 2-h post-load (F) time points. The increased and decreased metabolites are represented as red or blue scales, respectively. G and H: Venn diagrams of the overlapping metabolites detected between the two groups at separate time points; the increased metabolites are shown in G, and the decreased metabolites are shown in H. The significance was determined using *P*-values adjusted by the Benjamini-Hochberg method for either Student's *t*-test or Mann-Whitney test. Abbreviations: PCA, principal component analysis; PLS-DA, partial least squares discriminant analysis.

To explore metabolic differences between the two groups, we performed a comprehensive metabolomic analysis of the plasma from the subjects using UHPLC-Q-TOF-MS/MS. The overall distribution trends among all samples were observed using PCA analysis (***Fig. 2A*** and ***2B***). The PCA scores for the negative ion mode showed a significant separation between the two groups at different time points, while the positive ion mode displayed an insignificant trend. We performed PLS-DA to further differentiate the metabolite features and identify potential marker metabolites. The PLS-DA score plots showed well-distinguished differences between the groups at both time points in negative ion mode, with a partial overlap in positive ion mode at the fasting time point (***Fig. 2C*** and ***2D***).

Using the VIP values from the PLS-DA model (threshold > 1), a total of 164 metabolites exhibited differences between the two groups at separate time points (***Supplementary Fig. 1***). Metabolites with VIP > 1 were selected to construct hierarchical cluster plots for samples within each group, which were then used to evaluate the stability of the expression patterns of the selected target metabolites. Metabolites within similar clusters were presumed to share expression patterns, potentially indicating the presence of corresponding reaction steps in the metabolic pathways.

Taking into account the *P*-value from the Student's *t*-test (*P* < 0.05), at the fasting time point, the levels of 55 metabolites were significantly different between the control and the prediabetes groups (***Table 2*** and ***Fig. 2E***). Specifically, the levels of 20 metabolites, such as isocitrate, gamma-aminobutyric acid, and L-glutamic acid, were significantly increased, while other 35 metabolites, such as cholic acid, β-alanine, and L-proline, were significantly decreased in the prediabetes group, compared with the control group. At the 2-h post-load time point, 58 metabolites exhibited significant expression differences in the prediabetes group, compared with the control group (***Table 2*** and ***Fig. 2F***), among which, the levels of 39 metabolites, such as L-leucine, pyruvate, and chenodeoxycholic acid, were significantly increased, while other 19 metabolites, such as cholic acid, L-glutamine, and fumarate, were significantly decreased.

**Table 2:** Metabolites detected between the groups at different time points

Metabolites	Fasting		Metabolites	Two-hour post-load	
	log_2_(FC)	*P*-value		log_2_(FC)	*P*-value
(E)-Cinnamaldehyde	0.64	**	(R)-Carnitine	0.36	**
(S)-Allantoin	0.15	*	1,3,7-Trimethyluric acid	−0.22	*
(S)-Lactate^a^	−0.35	***	11-Deoxyprostaglandin E1	−0.55	*
1,3,7-Trimethyluric acid	−0.24	**	2(5H)-Furanone	0.21	***
10-Hydroxydecanoic acid	0.25	*	2-Butynedioic acid	0.03	*
11-Deoxyprostaglandin E1	−0.35	**	2-Deoxy-D-glucose^a^	−0.31	***
2-Aminoacrylic acid	−0.17	**	2-Oxoadipic acid	0.15	***
2-Deoxy-D-glucose^a^	−0.16	**	5,6-Dihydroxyindole	−0.35	***
3,4-Dihydroxymandelic acid	0.09	*	5-Acetamido-6-formamido-3-methyluracil	0.49	***
4-Hydroxyphenylacetic acid	0.12	*	7-Methylxanthine	0.23	*
7,9-Dihydro-1H-purine-2,6,8(3H)-trione	−0.28	**	Acetoin	0.11	*
7-Methylxanthine	−0.25	*	Acrylate	0.21	***
Allantoin	−0.43	**	Allantoin	−0.40	*
Alpha-ketocaproic acid	0.21	*	Alpha-D-galactose	0.22	***
Arachidonic acid	0.44	***	Benzene-1,2,4-triol	0.23	***
Benzoic acid	0.17	**	Butane-2,3-dione	0.25	***
Beta-alanine	−0.45	**	Chenodeoxycholic acid^b^	0.24	*
Cholic acid^b^	−0.32	*	Cholic acid^b^	−0.27	***
D-Aspartic acid	−0.49	***	Corticosterone	0.24	**
D-Glucono-1,5-lactone	−0.48	***	Cortisone	0.25	*
Dodecanedioic acid	0.42	*	Decanoate	0.38	*
Dodecanoate	−0.56	*	Decanoic acid	−0.28	***
D-Pyroglutamic acid^c^	0.19	**	D-Fructofuranose 6-phosphate	0.14	**
D-Tagatopyranose	−0.37	**	D-Glucuronate	0.16	*
dUMP	−0.63	***	D-Pyroglutamic acid^c^	0.07	*
Equol	0.34	*	dUMP	−0.25	*
Estrone	0.60	*	Fumarate^a^	−0.15	***
Fumarate^a^	−0.07	*	Galactose	0.51	***
Fumaric acid	−0.24	**	Glutaric acid	0.17	***
Gamma-aminobutyric acid^c^	0.09	**	Glycine betaine	0.10	*
Homovanillic acid	−0.06	**	Glycocholic acid^bc^	−0.20	*
Hyocholic acid^b^	0.25	*	Glycolate	0.28	***
Hypoxanthine	−0.66	***	Glycolic acid	−0.46	***
Isocitric acid^a^	0.23	**	Glyoxylic acid	0.26	***
L-Glutamic acid^c^	0.26	**	Hyocholic acid^b^	0.72	*
Linoleate	0.23	*	Hyodeoxycholic acid	0.12	*
L-Proline	−0.23	***	Indol-3-ylacetaldehyde	0.61	***
L-Rhamnose	−0.11	*	Indole-3-acetic acid	0.35	***
L-Threonic acid	−0.44	***	L-Arabinitol	0.13	***
L-Tyrosine	−0.35	**	L-Glutamine^c^	−0.08	*
Maltose	−0.44	***	L-Leucine	0.28	**
Methylmalonic acid	−0.51	***	L-Rhamnose	−0.44	***
N-Acetyl-L-alanine	0.22	*	L-Sorbose	0.23	***
N-Acetylneuraminic acid	−0.28	**	Methionine sulfoxide	0.13	*
N-Benzoylglycine	−0.23	*	Methylmalonate	0.17	***
N-Cinnamoylglycine	−0.08	**	Myo-inositol	0.30	***
Octadecanoic acid	0.21	**	N-Acetyl-L-proline	0.24	***
Octanoic acid	0.17	*	N-Acetylneuraminic acid	−0.38	*
Oxomalonic acid	−0.15	*	N-Cinnamoylglycine	−0.18	***
p-Hydroxyhippuric acid	−0.41	***	Octanoic acid	0.39	**
Purine	−0.49	*	Oxomalonic acid	−0.19	***
Pyridoxal	−0.51	*	Pyruvate^a^	0.26	***
Sucrose^a^	−0.35	***	Quinolin-2-ol	−0.27	***
Uric acid	−0.51	***	S-Sulfo-L-cysteine	0.29	*
Xylitol	−0.21	*	Theobromine	0.32	***
			Thymidine	−0.51	***
			Tyramine sulfate	0.15	**
			Xylitol	−0.29	**
^a^Metabolites associated with energy metabolism. ^b^Metabolites associated with bile acid metabolism. ^c^Metabolites associated with glutamate metabolism.Concentration changes of metabolites are log-transformed fold change (log_2_[FC]). The significance was determined using *P*-values adjusted by the Benjamini-Hochberg method for either Student's *t*-test or the Mann-Whitney test. ^*^P < 0.05, ^**^P < 0.01, and ^***^P < 0.001.					

Notably, compared with the control group, 15 metabolites in the prediabetes group showed consistent change trends at both the fasting and 2-h post-load time points, which included three consistently increased metabolites (D-pyroglutamic acid, hyocholic acid, and octanoic acid) and 12 consistently decreased metabolites (1,3,7-trimethyluric acid, 11-deoxy prostaglandin E1, 2-deoxy-D-glucose, allantoin, cholic acid, dUMP, fumarate, L-rhamnose, N-acetylneuraminic acid, N-cinnamoylglycine, oxomalonic acid, and xylitol; ***Fig. 2G*** and ***2H***).

### Metabolic pathway analysis

**Figure 3:**
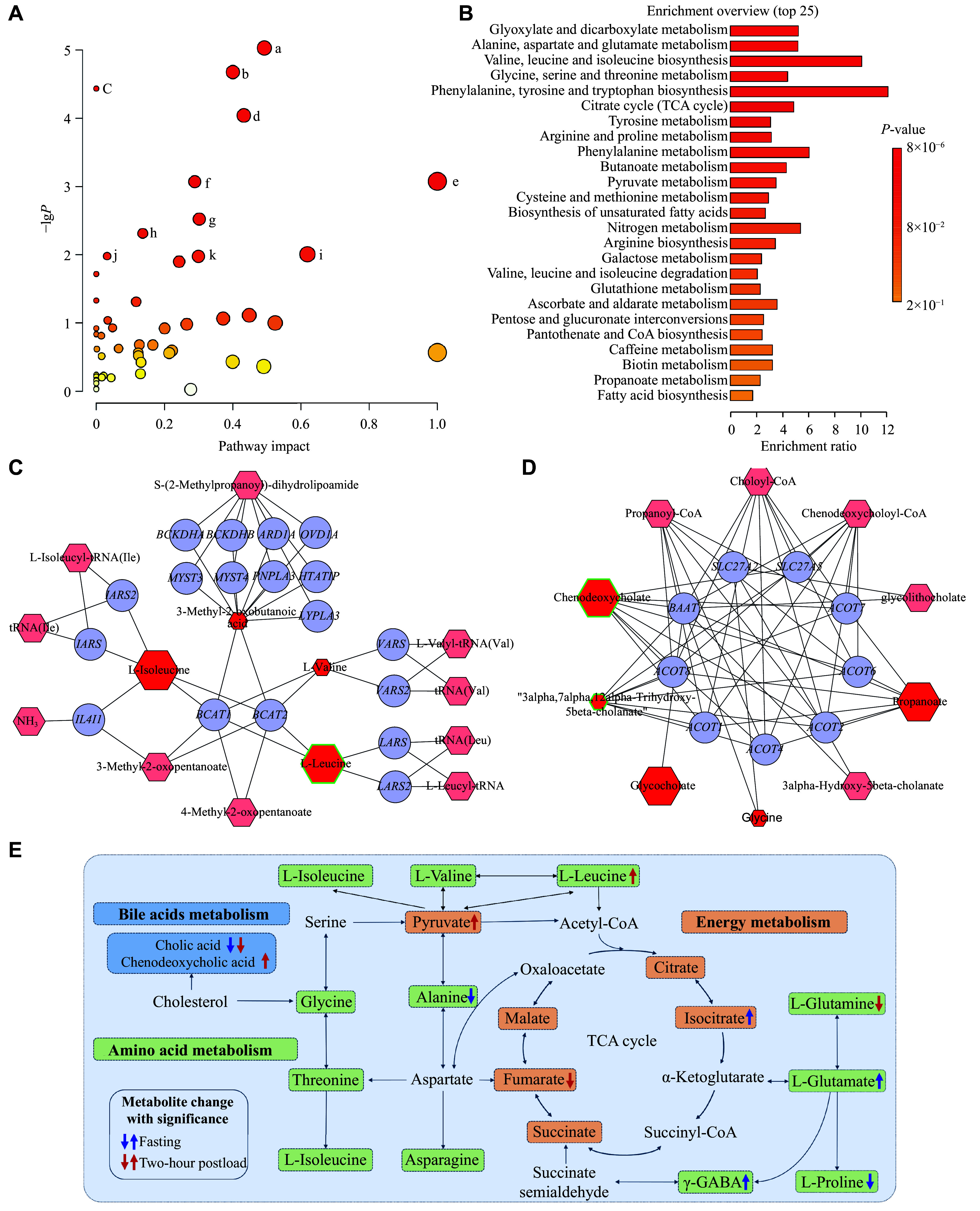
Metabolic profiling of pathway analysis, post-load interaction network, and the gestational diabetes mellitus to prediabetes progression pathways. A: Bubble graph of metabolic pathway analysis. (a) glyoxylate and dicarboxylate metabolism; (b) alanine, aspartate, and glutamate metabolism; (c) valine, leucine, and isoleucine biosynthesis; (d) glycine, serine, and threonine metabolism; (e) phenylalanine, tyrosine, and tryptophan biosynthesis; (f) citrate cycle; (g) tyrosine metabolism; (h) arginine and proline metabolism; (i) phenylalanine metabolism; (j) butanoate metabolism; (k) pyruvate metabolism. B: Bar graph of metabolite set enrichment analysis. C and D: Network of interactions between metabolites and genes at the 2-h post-load time point. C shows the valine, leucine, and isoleucine metabolism. D shows the bile acid biosynthesis. Hexagonal and circular nodes represent metabolites and genes, respectively. Red-colored hexagons indicate differential metabolites with variable importance in projection (VIP) >1. The largest hexagons denote an increase, while the smallest denote a decrease. Those with green borders indicate a comparison between the two groups with *P* < 0.05. Pink-colored hexagons represent metabolites associated with the pathways. E: Integrated metabolic pathways correlated with progression from gestational diabetes mellitus to prediabetes. Abbreviation: TCA, tricarboxylic acid.

Based on the results of PLS-DA, we entered differential metabolites with VIP > 1 into the MetaboAnalyst 6.0 platform for metabolic pathway analysis and metabolite set enrichment analysis between the two groups. ***Fig. 3A*** and ***3B*** display the main enriched metabolic pathways. The metabolite set enrichment analysis revealed significant enrichment in amino acid metabolism pathways and energy metabolism pathways, including alanine, aspartate, and glutamate metabolism, and other metabolic pathways that were closely related to disorders of glucose metabolism. Additionally, we employed Cytoscape software to construct a network of interactions between the differential metabolites and their upstream or downstream genes. A network diagram illustrating these interactions between the metabolites and genes was also generated (***Fig. 3C*** and ***3D***). These metabolites with VIP > 1, which were involved in various pathways, including those shown in ***Fig. 3E***, provided a sensitive reflection of metabolic abnormalities in the prediabetes group compared with the control group.

Leucine is involved in the metabolism of branched-chain amino acids (BCAAs), and the elevated levels of BCAAs are closely associated with the development of insulin resistance and type 2 diabetes^[15]^. The accumulation of pyruvate, which is involved in pyruvate metabolism, may trigger inflammation, thereby impairing the insulin signaling pathway. Fumarate and isocitrate are key intermediates of the tricarboxylic acid (TCA) cycle, and the impaired TCA cycle flux in insulin-resistant skeletal muscle is one of the characteristics of the diabetic phenotype.

### Prediction for the progression from GDM to prediabetes

**Figure 4:**
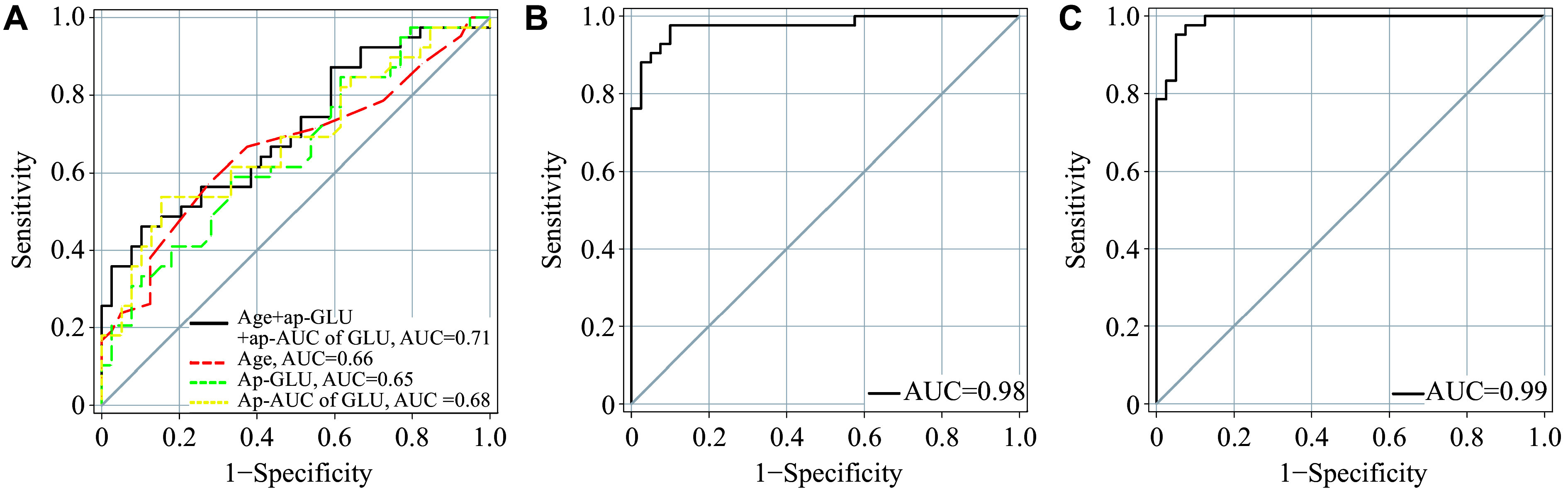
Receiver operator characteristic (ROC) curves. A: ROC curves of predictive models for the progression from gestational diabetes mellitus to prediabetes generated by age, 2-h post-load glucose levels, and the AUC of glucose during the pregnancy oral glucose tolerance test. B and C: ROC curves for the discriminative power of the panel of 15 metabolites for distinguishing between postpartum prediabetes and normal glucose tolerance at the fasting (B) and 2-h post-load (C) time points. Abbreviations: ap, antepartum; GLU, glucose; AUC, area under the curve.

Using the ROC curve, we assessed the predictive abilities of three clinical markers during pregnancy for the progression from GDM to prediabetes, including age, 2-h post-load glucose levels, and the AUC of glucose levels during the pregnancy OGTT (***Fig. 4A***). The AUCs of the three clinical markers ranged from 0.65 to 0.68. The model combined these three clinical markers, yielding an AUC of 0.71 (95% CI, 0.60–0.82), with a sensitivity of 0.46 and a specificity of 0.90 at the optimal cutoff. However, this increase was not statistically significant.

Given that the 15 metabolites in prediabetes showed consistent change trends before and after glucose loading, we also assessed the discriminative power of the panel of 15 metabolites for distinguishing between postpartum prediabetes and normal glucose tolerance at the fasting and 2-h post-load time points (***Fig. 4B*** and ***4C***). We achieved an AUC of 0.98 (95% CI, 0.94–1.00), a sensitivity of 0.95, and a specificity of 0.92 at the fasting time point, and an AUC of 0.99 (95% CI, 0.97–1.00), a sensitivity of 0.95, and a specificity of 0.95 at the 2-h post-load time point.

## Discussion

Prediabetes is a heterogeneous disorder of glucose metabolism characterized by IFG and/or IGT based on a 2-h glucose load. Postpartum follow-up based on OGTT is complicated and depends on blood glucose levels at fasting and 2-h post-load time points for the diagnosis of prediabetes.

In the current study, we conducted an OGTT-based follow-up of women with a history of GDM between six weeks and six months postpartum. Forty subjects had normal postpartum glucose tolerance, and 42 subjects were diagnosed with postpartum prediabetes, including seven with IFG, 31 with IGT, and four with both IFG and IGT. Plasma metabolite levels were significantly different between the control and prediabetes groups at both the fasting and 2-h post-load time points. The consistent detection of 15 differential metabolites in the prediabetes group at both the fasting and 2-h post-load time points highlights their potential utility in assessing disease conditions and identifying biomarkers for diagnosis and postpartum follow-up screening. The metabolomic pathway analysis provides insights into potential pathophysiological mechanisms and etiological factors underlying prediabetes in the early postpartum period.

Consistent with several international studies^[16–17]^, the current study found that the subjects were older in the prediabetes group than those in the control group. Advanced maternal age is a known predictor for abnormal glucose tolerance between six and 12 weeks postpartum^[18–19]^. We also observed that the subjects in the prediabetes group showed higher 2-h glucose values and the AUC of glucose during the pregnancy OGTT, compared with those in the control group, consistent with the findings from a systematic review by Moore *et al*^[20]^, in which a higher 2-h glucose level during pregnancy increased the risk of developing diabetes fourfold.

Previous studies on predicting the development of prediabetes and/or T2D have analyzed clinical markers and maternal serum or plasma metabolome during pregnancy or collected postpartum^[9–11,21]^. From a clinical perspective, there is a need to use only clinical markers that are easy to obtain during pregnancy to predict which women with a history of GDM are more likely to develop prediabetes postpartum. For example, Liu *et al*^[10]^ found that the addition of metabolites at approximately 28 weeks of gestation showed little improvement in predicting a postpartum disorder of glucose metabolism, compared with clinical factors alone. The AUC values of the clinical variables reported in previous studies^[9–11]^ were also similar (ranging from 0.68 to 0.745) to those observed in the current study (0.71). In contrast, another study^[21]^ with a small sample size and a longer follow-up period of nine years reported a slightly lower AUC for clinical markers (0.65). These results are relatively similar, notwithstanding the variations in the study populations, methodologies, and the clinical markers included. Therefore, clinical markers during pregnancy can be employed to screen for individuals at high risk of prediabetes preliminarily.

The combination of metabolites and clinical markers is expected to achieve a higher discriminating power than either used alone. Wang *et al*^[11]^ identified that lipid species at 24 to 72 h after delivery modestly improved the predictive performance for incident T2D (AUC_max_ ≤ 0.83) above classical risk factors across the first 15 years of follow-up. An American team, using the Study of Women, Infant Feeding, and Type 2 Diabetes after GDM Pregnancy (SWIFT) cohort and employing metabolomics and lipidomics, has established a series of predictive models for the progression from GDM to T2D, such as a panel of 12 lipids (AUC, 0.84)^[22]^, a panel of 11 lipids (AUC, 0.739)^[23]^, a signature of 20 metabolites (AUC, 0.88)^[9]^, and a model with 10 analytes (AUC, 0.78)^[24]^. Given the cross-sectional study, we found that a panel of 15 metabolites showed a robust discriminative power for distinguishing between postpartum prediabetes and normal glucose tolerance at both the fasting and 2-h post-load time points. However, future studies are warranted to validate the predictive power.

The metabolic profiling here revealed that energy metabolism and BCAAs played a role in the development of prediabetes in women with a previous history of GDM, which may provide insights into the underlying pathophysiology of the transition from GDM to prediabetes in the early postpartum period through the metabolic approach.

Both glycolysis and the TCA cycle disruption characterize diabetes in multiple tissues^[25]^. The impaired insulin signaling may trigger inflammation through pyruvate accumulation^[26–27]^. The impaired TCA flux in insulin-resistant human skeletal muscle is one of the characteristics of the diabetic phenotype^[28]^. Patients with normal blood glucose but insulin resistance exhibit a 32% reduction in ATP production, compared with the control group^[29]^. At the 2-h post-load time point, we observed an increase in pyruvate levels in the prediabetes group, compared with the control group, while the TCA product fumarate decreased without changes in other intermediates. These findings imply that the excessive post-load glycolysis and possibly impaired TCA under insulin resistance may disrupt energy metabolism and elevate glucose.

Furthermore, plasma BCAA levels are correlated with insulin resistance^[15,30]^, GDM^[31]^, prediabetes^[15]^, and T2D^[9,15]^. We previously identified elevated BCAAs before and after delivery in women with GDM using ^1^H-NMR^[32]^. The SWIFT cohort study observed that elevated BCAA levels at six to nine weeks postpartum in women with GDM were strongly associated with an increased risk of developing T2D in the future^[9]^. In addition, Andersson-Hall *et al*^[33]^ found higher fasting leucine levels in women with IGT than those with normal glucose tolerance at six years postpartum after GDM. In partial agreement with these findings, here we also found that leucine levels significantly increased at the 2-h post-load time point in the prediabetes group, compared with the control group. Leucine is a potent activator of mTORC1, and persistent activation of mTORC1 may lead to or exacerbate insulin resistance^[34–35]^.

The main strength of the current study lies in the identification of a panel of 15 metabolites that may distinguish between prediabetes and normal glucose tolerance in the early postpartum period in women with a history of GDM and could be considered for clinical application once validated in future studies.

The current study has some limitations. Our results may not be robust because of a small sample size (*n* = 82). The cross-sectional approach precludes establishing causal links between the development of prediabetes and metabolic variations. Future larger cohorts will help to validate these findings with long-term effects.

In conclusion, the current study highlights the metabolic changes associated with the transition from GDM to prediabetes in the early postpartum period, with a focus on energy metabolism and branched-chain amino acids. The identification of the panel of 15 metabolites with strong discriminative power for postpartum prediabetes suggests the potential for developing a metabolic profiling test for early risk assessment. While our findings require validation in larger, longitudinal studies, they provide a foundation for future research aimed at improving postpartum screening and early intervention strategies for women with a history of GDM. Such metabolic profiling could complement or potentially replace the current OGTT-based follow-up, addressing the challenges of low compliance and offering more precise risk stratification.

## SUPPLEMENTARY DATA

Supplementary data to this article can be found online.
