# Supplementary material for: Metabolic profiling identifies potential biomarkers associated with progression from gestational diabetes mellitus to prediabetes postpartum
Source: J Biomed Res. 2024 Nov 8;39(4):394–406. doi: 10.7555/JBR.38.20240267 (PMC12329413; doi:10.7555/JBR.38.20240267)
Supplement: Supplementary file 1 — Supplementary data to this article can be found online. [file jbr-39-4-394-Supplmentary.pdf]

Available online at www.jbr-pub.org.cn

Open Access at PubMed Central

JBR

Journal of Biomedical Research, 2025 39(4): 1–1

*Supplementary Data*

# Metabolic profiling identifies potential biomarkers associated with progression from gestational diabetes mellitus to prediabetes postpartum

Lenan Liu[1,2], Qian Yang[2], Panyuan Shen[2], Junsong Wang[3], Qi Zheng[3], Guoying Zhang[2,✉], Bai Jin[2,✉]

[1]*School of Public Health, Nanjing Medical University, Nanjing, Jiangsu 211166, China;*
[2]*Department of Obstetrics, the First Affiliated Hospital of Nanjing Medical University, Nanjing, Jiangsu 210029, China;*
[3]*Center of Molecular Metabolism, Nanjing University of Science and Technology, Nanjing, Jiangsu 210094, China.*

***Supplementary Fig. 1*** (available online) shows the heat map analysis of metabolites. The heat map displays 164 identified differential metabolites in each group (A) or sample (B). The values of differential metabolites were normalized and are shown on a color scale. The high and low metabolite levels are represented by red and blue or green scales, respectively. The significance was determined using *P*-values adjusted by the Benjamini-Hochberg method for either Student's *t*-test or the Mann-Whitney test. $^{*}P < 0.05$, $^{**}P < 0.01$, and $^{***}P < 0.001$, comparing the difference between the control ($n$ = 40) and prediabetes ($n$ = 40) groups at the fasting time point. $^{\#}P < 0.05$, $^{\#\#}P < 0.01$, and $^{\#\#\#}P < 0.001$, comparing the difference between the prediabetes and control groups at the 2-h post-load time point.

✉Corresponding authors: Guoying Zhang and Bai Jin, Department of Obstetrics, the First Affiliated Hospital of Nanjing Medical University, 300 Guangzhou Road, Nanjing, Jiangsu 210029, China. E-mails: 1149881120@qq.com (Zhang) and jinbai1018@yeah.net (Jin).

Received: 26 August 2024; Revised: 22 October 2024; Accepted: 24 October 2024; Published online: 08 November 2024

CLC number: R714.256, Document code: A

The authors reported no conflict of interests.

This is an open access article under the Creative Commons Attribution (CC BY 4.0) license, which permits others to distribute, remix, adapt and build upon this work, for commercial use, provided the original work is properly cited.

© 2025 by Journal of Biomedical Research.

https://doi.org/10.7555/JBR.38.20240267
